# Is it too complex? A survey of pediatric residency program’s educational approach for the care of children with medical complexity

**DOI:** 10.1186/s12909-023-04324-y

**Published:** 2023-05-12

**Authors:** Kira Sieplinga, Christopher Kruger, Emily Goodwin

**Affiliations:** 1 Michigan State University College of Human Medicine, 15 Michigan St NE, Grand Rapids, MI 49503 USA; 2 Department of Pediatrics, Spectrum Health Helen DeVos Children’s Hospital, 100 Michigan St NE MC 188, OME Suite Office #A624, Grand Rapids, MI 49503 USA; 3 Department of Pediatrics, University of Michigan C.S. Mott Children’s Hospital, 1540 E Hospital Dr, Ann Arbor, MI 48109 USA; 4 Division of General Academic Pediatrics, Children’s Mercy Kansas City, University of Missouri Kansas City School of Medicine; University of Kansas School of Medicine, 2401 Gillham Rd, Kansas City, MO 64108 USA

**Keywords:** Children with medical complexity, Graduate medical education, Pediatric residents

## Abstract

**Background:**

Although Entrustable Professional Activities (EPAs) regarding pediatric training in care for children with medical complexity (CMC) exist, it is unknown what US pediatric training programs provide for education related to care of CMC and whether educators perceive that pediatric residents are prepared to care for CMC upon graduation.

**Methods:**

From June, 2021 through March 2022, we surveyed US pediatric residency program delegates about practice settings, current educational offerings, perception of resident preparedness regarding care of CMC, and likelihood to implement CMC education in the future.

**Results:**

Response rate was 29% (56 /195). A third of responding programs (34%, *n* = 19) provide a specific educational CMC offering including combinations of traditional didactics (84%, *n* = 16), asynchronous modules/reading (63%, *n* = 12), experiential learning (58%, *n* = 11), and simulation-based didactics (26%, *n* = 5). The majority (93%, *n* = 52) of respondents agreed residents should be competent in providing primary care for CMC upon graduation and CMC should receive primary care from a resident (84%, *n* = 47). A total of 49% (*n* = 27) of respondents reported their residents are very or extremely well prepared to care for CMC after graduation. A total of 33% (*n* = 18) of programs reported CMC receive primary care from residents. Respondent average perception of resident preparedness was significantly higher in programs with educational offerings in five of eleven EPAs (nutrition and weight, transitions, feeding tubes, advocacy, and care coordination). The majority (78%, *n* = 29) of programs without educational offerings are at least somewhat likely to implement CMC curricula in the next three years.

**Conclusion:**

Pediatric residency programs report residents should be competent in care for CMC upon graduation. Pediatric residents are exposed to a wide variety of clinical care models for CMC. The minority of responding programs have intentional CMC educational offerings. Of those programs that provide CMC education, the offerings are variable and are associated with a perception of improved preparedness to care for CMC upon graduation.

**Supplementary Information:**

The online version contains supplementary material available at 10.1186/s12909-023-04324-y.

## Background

Children and youth withspecial health care needs (CYSHCN) have been identified as a priority population by the Institute of Medicine (IOM) since 2001 [1]. Over the past decade, children with medical complexity (CMC) have emerged as an important subset of this population. CMC have multiple chronic conditions, functional limitations, high utilization of health care use and high impact on their caregivers and families [2–5]. Although CMC represent less than 1% of the pediatric population, they account for at least one third of health care expenditures and thus, various models of care have emerged to care for this population [6]. In describing the multiple models of care, Galligan and Hogan describe lack of standardized care models as a “Goldilocks problem” and one that requires individual healthcare systems to find a complex care model that suit their own health system and its needs without any specific ‘gold standards’ to outline core program requirements. These acute and chronic models of care include (but are not limited to) primary care-centered models, consultative models, and episode-based models implemented across United States children’s hospitals and medical centers [7].

Given the high interaction of CMC with the health care system, is very likely that pediatric residents will encounter CMC during training and in practice. It is unclear how current pediatric residency training programs prepare residents to care for CMC and how residents are exposed to model(s) of care for CMC. Previous studies found that pediatric residents experience fear and discomfort in caring for CMC [8–10]. Given a wide variety of care models, this fear and apprehension may come from lack of effective training [10] and lack of standardized educational exposure.

*The Accreditation of Council for Graduate Medical Education*(ACGME) mandates that a pediatric residency curriculum must provide the longitudinal management of children with special health care needs and chronic conditions [11]. Likewise, the American Board of Pediatrics has published an Entrustable Professional Activity (EPA) for provision of a medical home for patients with complex, chronic, or special health care needs [12]. While neither governing body yet mandates specific training in care of CMC, many different educational resources for care of CMC have emerged because of the growing interest in the field of CMC [13]. Similar to the multiple models of care found nationally, the published approaches to education of CMC are variable and include, amongst others, simulation-based education, rotational opportunities, traditional didactics, web-based modules and family-centered education [14–17]. In 2020, Huth et al. published a set of EPAs for care of CMC after sensing a need for consensus amidst a rapidly evolving and programmatically diverse field. [18] Building on the work of previous curricular needs assessments [19], eleven EPAs were identified through expert consensus using Delphi methodology. These EPAs include management of CMC with respect to feeding difficulties and nutrition, pain and irritability, motility, aspiration, neuromuscular/skeletal issues, safety/emergency planning, transition, feeding tubes, goals of care, advocacy, and team-based care coordination [18, 20].

Given the wide variety of clinical care models, a large body of educational resources and a consensus-driven set of EPAs for care of CMC, it is valuable to understand if and how pediatric residencies are currently providing education for their trainees in care of CMC. In 2010, Nazairan et. al discovered that pediatric residency programs indeed offer a variety of successful educational and clinical experiences related to the medical home and CYSHCN [21]. To our knowledge, few studies have determined the extent and type of educational clinical exposure to care of CMC during pediatric residency training. Additionally, no studies have evaluated the current state of educational opportunities for CMC across general United States pediatric training programs. Further work is necessary to understand what curricular content and methodologies are being used to teach and prepare future pediatricians to care for CMC.

Our objective was to describe educational opportunities in US pediatric residency programs regarding care for CMC. Our secondary objectives were to assess pediatric residency leadership perceptions of resident preparedness to care for CMC with current training and likelihood of implementing formal CMC curricula.

## Methods

### Study participants

The survey was approved by the Association of Pediatric Program Directors (APPD) for distribution to associate program directors (APDs) from pediatric residency programs. APDs also had the option to choose a delegate from their program who may have a better understanding of the care setting and curricula offered within their specific learning environment.

### Survey construction

We designed the survey to measure our objectives and utilized the Entrustable Professional Activities in Complex Care as a framework [18, 20]. We applied Messick’s validity framework [22] to this survey using the following strategies: two expert contributors from the field of complex care were contacted to develop survey items and provide expert content review and cognitive interviews were conducted with several pediatric residency program directors to validate response process. The survey was evaluated for common survey pitfalls including use of construct-specific response items and avoidance of multi-barreled items before submission [23]. The survey was IRB exempted, peer reviewed, and accepted for distribution in 2021 by the APPD Research and Scholarship Learning Community after modification suggestions were made by an expert panel from the APPD.

### Survey content

The survey contained thirty total questions (see supplemental materials). Initial questions assessed curricula and other educational offerings for residents involving care for CMC. Ensuing questions inquired about perceptions of resident preparedness to care for CMC based on core curricular EPAs for care for CMC [18, 20]. Participants rated resident preparedness on eleven EPA priority topics using a 5-point Likert scale: 1 = not at all prepared through 5 = extremely prepared. Final questions assessed programs’ likelihood to implement formal CMC curricula and potential barriers to implementation.

### Survey administration

Using Lime survey software [24], APPD distributed the survey to APDs who, if needed, could delegate completion to alternative faculty. The survey remained open for eight weeks from June to September of 2021 during which time APDs received one invitation and five weekly reminders. Distribution of the survey followed the APPD research and learning community standard procedures which includes initially distributing to one APD. Per APPD distribution policy, after the initial period, sites that had not responded were attempted to be reached through a second listed APD with one invitation and three reminders. Once a reply was received from a single institution, that unique survey was closed thus eliminating the possibility of receiving two unique responses from the same institution. Initial response rate was lower than anticipated (15%, *n* = 29) therefore APPD re-opened the survey in March of 2022 for two weeks and sent to all enrolled APDs using the same process. The survey closed after the second response period. Two institutions responded to the survey during both response periods. The chronologically older responses from those two programs were excluded from data analysis.

### Data analysis

We calculated summary and descriptive statistics for quantitative questions. With statistical support, we used Chi-Square and Fisher’s Exact tests to compare participant institution demographics to APPD demographics. Independence of survey responses based on presence or absence of curricula were analyzed using Fisher's Exact test for count variables and Kruskal–Wallis test for averaged variables. For statistical power, we combined the small and medium residency respondents into one group called “small” in order to compare small vs. large programs. Statistical significance was assessed at the *p* < 0.05 level. We excluded response options that were marked as 'unsure' from statistical analysis.

## Results

### Survey response rate and demographics

The complete survey response rate was 29% (56 unique program representatives of 195 total programs). Partial survey responses were not reported to investigators and thus not included in analysis. Demographics of respondent programs did not differ significantly from the APPD as a whole by region (*p* = 0.13) affiliation (*p* = 0.79), or size (*p* = 0.28) (Table 1). We did not determine whether the initially queried APD completed the survey, a second APD completed the survey or whether the survey was completed by a delegate; thus, each unique submission was classified as a “respondent.” The following results are organized by objective measured.

**Table 1:** Demographics of programs surveyed (*N* = 56). Overall response rate was 56/195 (29%). Demographics of respondent program did not differ significantly from APPD as a whole, including by region (*p* = 0.13) affiliation (*p* = 0.79), and size (*p* = 0.28)

AAPD Region	#(%)	Institution Type	#(%)	Program Size	#(%)
Mid-America	5(17)	Community-based	2(7)	Small (< 30)	4(14)
Mid-Atlantic	1(3)	Community-based, university affiliated	13(45)	Medium (30–70)	17(59)
Midwest	2(7)	Military	1(3)	Large (> 70)	8(27)
New York	4(14)	University based	13(45)		
Northeast	2(7)				
Southeast	6(21)				
Southwest	4(14)				
Western	5(17)				

#### Objective 1: to describe current educational opportunities amongst national general pediatric residency programs

The survey was designed to determine where CMC are cared for within the learning environment and how residents were exposed to this patient population. In addition, we sought to determine whether specific CMC educational offerings or clinical exposure opportunities were provided.

##### Patient care locations

Primary care for CMC is provided at 98% (*n* = 55) of residency continuity clinics. In addition to existing continuity clinics, 55% (*n* = 31) of responding programs have specific outpatient clinics to care for CMC that are distinctive from the residency continuity clinic. A total of 20% (*n* = 11) of responding programs have CMC-specific inpatient teams. We did not determine whether programs that have a dedicated inpatient service also have a dedicated specific outpatient clinic. In comparison of small (70 or less residents, *n* = 12) vs large (71 or more residents, *n* = 44) programs, there were no associations between provider of care (Advanced Practice Provider vs Attending vs Resident) for CMC (*p* = 0.79), specific outpatient clinics to care for CMC (*p* = 0.62) or CMC-specific inpatient teams (*p* = 0.22).

##### Clinical Exposure

The majority (84%, *n* = 47) of respondents report that CMC should receive primary care from a resident. A total of 35% (*n* = 20) of respondents intentionally assign CMC to resident panels at their continuity clinics and most CMC receive their primary care from attending physicians (65%, *n* = 36) or advanced practice providers (2%, *n* = 1). Of the 11 programs with inpatient teams dedicated to CMC, nine require residents to rotate for at least two weeks on the CMC service. A total of 31% (*n* = 17) of respondents provide at least two weeks of outpatient rotations caring for CMC.

**Fig. 1:**
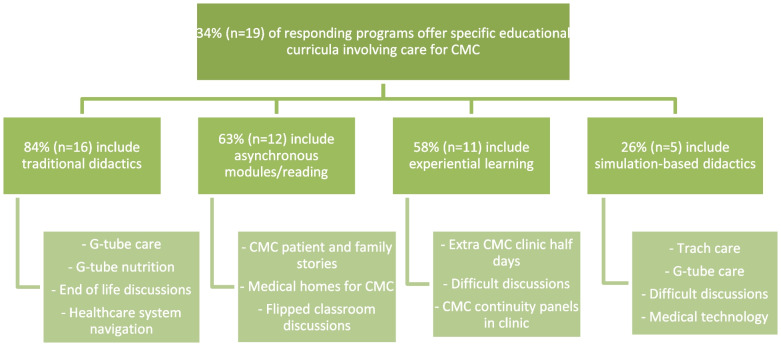
CMC educational offering breakdown with specific examples from free response prompts

Additional educational opportunities: A total of 34% (*n* = 19) of respondents provide specific educational opportunities involving care for CMC. These opportunities include combinations of traditional didactics (84%, *n* = 16), asynchronous modules/reading (63%, *n* = 12), experiential learning (58%, *n* = 11), and simulation-based didactics (26%, *n* = 5). Figure 1 demonstrates free text responses from respondents further characterizing their offerings. The survey was not designed to evaluate these responses qualitatively; however, anecdotally, the offerings varied widely. Selected descriptions of educational offerings included:

“We have noon conference didactics and case-based conferences inclusive of topics related to CMC”

“We have developed a module on caring for children with medical complexity that is delivered as a flipped classroom with asynchronous reading and an in-person discussion once per year.”

“All interns have a 3-hour workshop that...uses flipped classroom and an Open Pediatrics module that focuses on the story of a patient family.”

“Simulations are part of…hands on training…. including G-Tube care, tracheostomy replacement.”

“The residents receive at least one noon conference lecture specifically discussing the care of CMC.”

#### Objective 2: to determine pediatric residency leadership perceptions of resident preparedness to care for CMC with current training

Over 93% (*n* = 52) of respondents agreed that pediatric residents should be competent in providing primary care for CMC upon graduation. There was a difference in small vs large program’s respondent perceptions of whether graduating residents should be competent in primary care for CMC. Large program respondents had a higher level of agreement (4.72) that graduating residents should be competent in primary care for CMC than small program respondents (4.08, *p* = 0.045).

**Fig. 2:**
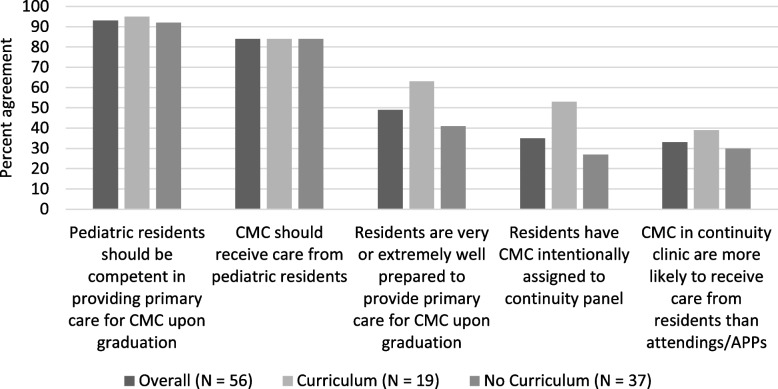
Program director perceptions and resident continuity opportunities compared by presence of CMC curricula

Respondent perception of resident preparedness to provide primary care for CMC upon graduation from residency is shown in Fig. 2. The term “preparedness” was chosen as residents are asked how well prepared they feel at the end of residency by the ACGME and thus the term preparedness was thought to be familiar to respondents. Overall, 48% (*n* = 27) of respondents felt as if graduating residents are very (37%, *n* = 21) or extremely (11%, *n* = 6) prepared to care for CMC upon graduation whereas 52% (*n* = 29) judged their residents to be somewhat (48%, *n* = 27), slightly (2%, *n* = 1), or not at all (2%, *n* = 1) prepared. We then asked respondents to rate resident preparedness for each specific EPA. In seven of eleven EPAs (advocacy, feeding tubes, difficult discussions, pain and irritability, transition, neuromuscular and skeletal symptoms, and emergency planning), a majority (> 50%) of programs rated graduating residents as somewhat, slightly, or not at all prepared. In four of eleven EPAs (feeding difficulties and nutrition, care coordination, managing dysmotility, and advocacy), a majority (> 50%) of respondents rated graduating residents as very or extremely prepared.

### Analysis of differences based on presence of educational opportunities

In a secondary analysis, we sought to understand if there is an association between responses to survey questions based on the presence or absence of CMC educational opportunities (phrased as curricula in the survey). The only association found was that respondents from programs with curricula perceived residents as very or extremely well prepared to provide primary care for CMC 22% more often than those without curricula (*p* = 0.16); otherwise, no associations were found with respect to clinical exposures and the presence or absence of curricula (see Fig. 2).

**Fig. 3:**
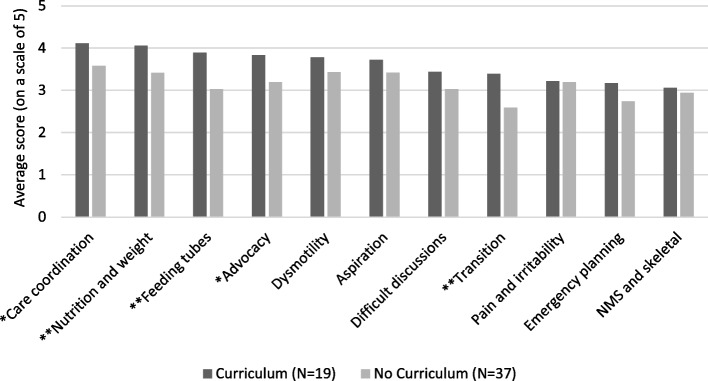
Average program representative perception of residents' preparedness for EPAs in core curricular topics of care for CMC compared by presence of curricula (*P < 0.05, **P < 0.01)

Perception of resident preparedness scores for individual EPAs were also stratified based on presence of curriculum as shown in Fig. 3. Average respondent perception of resident preparedness was higher for the curricula group in each of the eleven EPA categories. Five of these differences were statistically significant including nutrition and weight (*p* = 0.008), transitions (*p* = 0.006), feeding tubes (*p* = 0.003), advocacy (*p* = 0.028), and care coordination (*p* = 0.047).

#### Objective 3: to determine likelihood to implement formal CMC curricula 

Of the 37 residency programs without formal curricula, 78% (*n* = 29) are somewhat (31%, *n* = 11), very (39%, *n* = 14), or extremely (8%, *n* = 3) likely to implement a formal CMC curriculum within the next three years and 22% (*n* = 8) are slightly (17%, *n* = 6) or not at all (5%, *n* = 2) likely. There was no statistically significant difference between small and large programs with respect to likelihood of implementing CMC curricula within the next 3 years (*p* = 0.83). When asked to choose the top three factors that limit resident preparedness to care for CMC upon graduation (with option of free text “other” response), responses included: clinical time constraints, ability to integrate care for CMC into other topics, competing educational interests, lack of financial resources, and few faculty members to teach care for CMC.

## Discussion

Given the wide variety of clinical care settings for CMC and the large variety of educational resources to teach about care for CMC, it is important to understand the current landscape of clinical exposure and educational curricula for pediatric residents nationally.

### Objective 1: to describe current educational opportunities amongst national general pediatric residency programs.

Respondents report a wide variety of clinical care models in their learning environments. This finding is unsurprising as national care models also vary widely generally falling within one of three types of care models: (1) primary care-centered models, (2) consultative- or co-management-centered models, and (3) episode-based models [25]. The true distribution of programs is unknown and has not yet been published, but the Academic Pediatric Association Complex Care and Disability Special Interest Group has an affinity group that hosts a password protected member populated director of complex care programs including the United States and Canada, with 107 listed from the United States. Many but not all are hospital affiliated and programs vary on offerings of inpatient, outpatient, transitional care, short and long term care facilities, residential, school-based programs, consultative models or a mixture of these [26]. These self-reported 107 programs interact to an unknown extent with the approximately 10,000 currently active categorical pediatric trainees across 235 categorical pediatric residencies as of 2022 [27]. What is known and can likely be deduced from the information above is that despite attempts at creating more uniform care models for CMC, most CMC do not receive care in well-functioning health care systems [28]. It is also likely that many of the active pediatric residents do not train in a program supported by a well-functioning health-care system targeting CMC.

From an educational perspective, the national landscape can serve both as a challenge and as an opportunity. Amongst our respondents, only 35% of programs assign CMC to resident care panels. As healthcare systems work to design functioning systems to care for CMC, the desire to train pediatricians in care for CMC – particularly through continuity– should serve as an opportunity to inform decision makers. Given that some institutions have specific outpatient clinics and/or inpatient teams to care for CMC, it is possible that institutions recognize the need for specialized providers and teams to provide unique continuity for CMC and their families. Longitudinal relationships have been identified as feasible and important for learning to care for children with special needs, but this type of continuity may be logistically challenging during a traditional pediatric residency schedule and in programs with limited access to CMC [10]. It is also unknown whether continuity from resident physicians qualifies as a “well-functioning” healthcare system that serves the needs of the patients and their families. We would recommend learning from CMC caregivers and their desire to receive care from resident physicians in a continuity setting.

Thus, further study is needed to determine educational opportunities that do not solely rely upon continuous relationships with CMC to create preparedness upon graduation. This is particularly important for healthcare systems that rely upon episode-based or consultative models of care for CMC as not all pediatric residency programs have equal access to all models of care.

Given the lack of consistency of clinical exposures and care models, pediatric residency leadership will need to rely upon enhanced educational opportunities within training to assure competence of graduates. As evidenced by the variety of educational opportunities reflected in our results, educating about CMC during pediatric residency training has room to mature. It is encouraging that several respondents reported educational strategies that do correlate with the EPA framework [18] and that have been reported as effective in educational literature [8, 13–17]. Utilizing published flipped classroom strategies, simulation and incorporating elements of CMC EPAs into existing rotations such as inpatient rotations, neurodevelopmental rotations or electives are options to improve educational exposure [13]. These strategies may assist programs that are smaller, more community-based or do not have an established clinical care model for CMC within their institution.

In addition, we recommend that governing bodies such as the ACGME pediatric review committee consider specific program requirements focusing on education for CMC. The ACGME mandates specific training in topics such as adolescent medicine, critical care, and advocacy (amongst others) – why not also consider a mandate in topics related to CMC. Given that CMC account for up to one third of health care expenditures, health-care systems should also desire pediatricians who feel prepared and competent to care for this population.

### Objective 2: to determine pediatric residency leadership perceptions of resident preparedness to care for CMC with current training

The chosen term “preparedness” is subject to bias in reporting. Because the eleven published EPAs are relatively new and may not be known by many program directors nationally, determining preparedness is inherently subjective. However, the term is used by the ACGME when surveying program graduates and faculty of pediatric residencies. Thus, we posit that the term preparedness is familiar to respondents. Our respondents reported lack of preparedness of their graduates to care for CMC and to perform seven of eleven EPAs. This is concerning and likely reflects the lack of “well-functioning” health care models for CMC as well as general lack of awareness about educational resources to teach about CMC. These findings align with a recent study that shows pediatric residents themselves report limited self-entrustment in performing key clinical activities in complex care [29]. Next steps in understanding pediatric residents’ level of preparedness in caring for CMC would be to directly survey pediatric residents, recent pediatric residency graduates or employers of recent graduates.

### Objective 3: to determine likelihood to implement formal CMC curricula

Although respondents desire to implement educational opportunities in the next three years, time, expertise, and resources serve as barriers. Programs that desire to enhance education but have limited time could choose to focus opportunities targeted at the CMC EPAs as in our findings, the presence of any educational offerings led to increased perception of preparedness in some of the EPAs.

Further research is needed to identify recommendations for more standardized exposure to care for CMC to achieve competence. When the IOM identified care for CYSHCN as a priority population, residencies responded by implementing successful curricula. This study can serve as a needs assessment of the current landscape and as a steppingstone for further work to garner resources to address barriers, analyze effectiveness of curricula, and implement nationally recognized CMC EPA and ACGME requirements. Implementation of standardized curricula regarding care for CMC and continuity may lead to increased resident confidence and knowledge in caring for CMC [13] and ideally, better patient outcomes.

Our study was limited by potential response biases, the subjective nature of the term preparedness and the response rate. The study was conducted during the COVID-19 pandemic when anecdotally survey response rates dropped, potentially from survey fatigue and time constraints. A response rate of 29% is consistent with other similar reported APPD survey studies with response rates ranging from 14 to 48% [30]. Our sample was statistically representative of the APPD program distribution as a whole and thus may represent first steps in understanding the current landscape for complex care training in pediatric residency nationally. However, although response did represent program distribution, it is possible that respondents who have increased knowledge of CMC or specific interest were more likely to respond; thus, over-estimating the proportion of educational offerings for CMC nationally. In addition, due to the low power of the study, we were unable to associate any demographics (aside from small vs. large program size) with results.

Despite limitations, we conclude that this survey demonstrates several key points and exposes need for further study. First, this study shows a wide variety of exposure to CMC amongst pediatric residencies and demonstrates a need for further work in defining standards and effectiveness of educational methods. Second, further work is needed to determine resident perception of their preparedness to care for CMC upon and after graduation. As discussed above, future directions may include surveys of residents and their perceived level of preparedness (similar to the current ACGME survey) with respect to the eleven EPAs upon which the framework of this survey was built. Additionally, graduates of programs could be queried regarding what educational offerings were truly practical and applicable in “real-life practice.” Third, resident involvement in care models at programs across the country is variable but should not be ignored. When structuring care models for CMC at pediatric training facilities, educational exposure should be considered.

This study provides further information about the current landscape of the learning environment with respect to care for CMC. We do not believe that training pediatric residents about CMC is “too complex,” but it may require standardization, intentionality, and attention to available educational resources.

## Supplementary Information

**Additional file 1: Supplemental Materials.** Original Survey.

**Additional file 2. **

**Additional file 3. **

## Data Availability

Original data is available as supplemental material in deidentified Excel and pdf formats. The survey tool is available as an appendix and has been submitted in the supplemental materials.

## Abbreviations

CMC: Children with Medical Complexity
APPD: Association of Pediatric Program Directors
APD: Associate Program Director
IOM: Institute of Medicine
CYSHCN: Children and Youth with Special Health Care Needs
EPA: Entrustable Professional Activities
ACGME: The Accreditation of Council for Graduate Medical Education
