# Supplementary material for: Is it too complex? A survey of pediatric residency program’s educational approach for the care of children with medical complexity
Source: BMC Med Educ. 2023 May 12;23:331. doi: 10.1186/s12909-023-04324-y (PMC10174732; doi:10.1186/s12909-023-04324-y)
Supplement: Supplementary file 1 — Additional file 1: Supplemental Materials. Original Survey. [file 12909_2023_4324_MOESM1_ESM.pdf]

Supplemental Materials – Original Survey

For the purposes of this survey, a child with medical complexity (CMC) will be defined as a child who has multiple significant chronic health problems that affect multiple organ systems and result in functional limitations, high health care need or utilization, and often the need for or use of medical technology (Kuo, et al 2011).

Objective 1: To describe current educational offerings for pediatric residents in the care of children with medical complexity

Question 1: The continuity clinic experiences for our residents are:

1. Located within a primary clinical site sponsored by our children's hospital or university system
2. Based in individual community preceptor practices
3. A combination of the above
4. Other (please explain)

Question 2: Does your residency continuity clinic site provide primary care for CMC?

A. Yes
B. No

If no to question 2, please proceed to question 3

****

If yes to question 2....

2a) In your residency continuity clinic, who is most likely to provide primary care for CMC?

1. Attending Physicians
2. Advanced Practice Providers
3. Pediatric Residents

2b) Do residents have CMC intentionally assigned to their continuity panel?

A. Yes
B. No
C. Unsure

2c) At which level of training are residents assigned CMC to their panel? (Select all that apply)

A. PGY1
B. PGY2
C. PGY3 or above

****

The following questions are related to care experiences for CMC at your institution.

Question 3: Does your institution have an outpatient clinic that provides primary care for children with medical complexity (distinctive from the residency continuity clinic or other subspecialty clinics)

A. Yes
B. No
C. Unsure

Question 4: Does your hospital system have a specific inpatient team to provide non-ICU care for children with medical complexity?

A. Yes
B. No
C. Unsure

If “no” or “unsure” to question 4, please proceed to question 5.

****

If yes to question 4....

4a) Do residents rotate on this service for children with medical complexity?

A: Yes, this is a required rotation for 2 weeks or less
B: Yes, this is a required rotation for 2-4 weeks.
B: Yes, but offered only as an elective option and is not required C: No

****

Question 5: Do you provide an outpatient rotation for caring for CMC?

A: Yes (2 weeks or less) B: Yes (2-4 weeks)
C: No

Question 6: Aside from rotational experiences, do your residents receive a specific educational curriculum to care for CMC?

A: Yes
B: No

****
If yes to question 6...

6a) Is your curriculum (choose all that apply):

A) Traditional Didactics
B) Simulation-based didactics
C) Asynchronous modules/reading
D) Based on experiential learning (for example: bedside teaching, immersion, intentional relationships)
E) Other (please explain)

6b) Optional: Please describe your curriculum and why you selected the categories in question 6a. Your explanation is valuable in helping us understand the variety of educational offerings across pediatric residencies. (Free text)

****

Objective 2: To determine pediatric residency program director perceptions of resident preparedness to care for children with medical complexity with current training

Question 7: When residents graduate from your program, how prepared are they to independently provide primary care for CMC?

1. Extremely prepared
2. Very prepared
3. Somewhat prepared
4. Slightly prepared
5. Not at all prepared

****
If not A or B.....
7a) Aside from time, what are factors that limit resident preparation to care for CMC upon graduation? (Please select the top three)

- Inadequate knowledge of specialized CMC clinical topics
- Resident lack of interest in caring for CMC
- Residents feeling overwhelmed as providers for CMC
- Preferences of caregivers of CMC
- Lack of expertise of faculty/preceptors in care of CMC
- No clear national competencies

- Low patient volume of CMC (limited experience opportunities)
- Other (fill in)

****

Question 8: In a recent Delphi study published in academic pediatrics, the following topics related to CMC were identified as essential by >70% of participants in all rounds and are in alignment with the international classification of functioning, disability, and health (ICF) domains. (Huth, et al; Academic Pediatrics, 2020) Please rate the preparedness of graduates from your programs on the following topics:

How prepared are your residents?	Extremely	Very	Somewhat	Slightly	Not at all	Unsure
1. Feeding difficulties and nutritional concerns (including decision-making for tube placement, poor weight gain and obesity)						
2. Pain and irritability (including evaluation and management)						
3. Transition (including access to services, medical team transition of care, employment, independent living)						
4. Feeding tube management and troubleshooting (including gastrostomy, gastrojejunostomy)						
5. Difficult discussions (including palliative care, DNR, developing shared goals of care)						
6. Team management and care coordination						
7. Dysmotility (including constipation, GERD, slow gastric emptying, feeding intolerance, and indications for subspecialty referral)						
8. Aspiration (including evaluation and management of dysphagia, sialorrhea, chronic lung disease, and when to refer)						
9. Safety/emergency planning (including development of sick plans and emergency letters/summaries, recognizing special circumstances that require individualized management)						
10. Common neuromuscular and skeletal issues (including basics about spasticity management, hip and spine surveillance, bone health)						
11. Advocacy for patients/families (including developing partnerships, identifying stressors and risk factors for caregiver burnout, and liaising with the interdisciplinary team, community agencies and school)						

Objective 3: To determine interests and attitudes towards implementing curriculum related to children with medical complexity

Question 9: Please indicate your agreement with the statement:

“Pediatric Residents should be competent in providing primary care for CMC upon graduation from residency.”

A. Strongly Agree
B. Agree
C. Disagree
D. Strongly Disagree

Question 10: If not mandated by the ACGME, how likely are you to implement a formal educational curriculum for CMC in the next 3 years?

1. Extremely likely
2. Very likely
3. Somewhat likely
4. Slightly likely
5. Not at all likely
6. N/A: our program already has a formal curriculum

Question 11: Should children with medical complexity receive primary care from a resident?

A. Yes
B. No
C. Unsure

Question 12: (Optional) Do you have any additional comments regarding implementation of curriculum for CMC or training in the care of CMC during pediatric residency?
