# Supplementary material for: Is it too complex? A survey of pediatric residency program’s educational approach for the care of children with medical complexity
Source: BMC Med Educ. 2023 May 12;23:331. doi: 10.1186/s12909-023-04324-y (PMC10174732; doi:10.1186/s12909-023-04324-y)
Supplement: Supplementary file 3 — Additional file 3. [file 12909_2023_4324_MOESM3_ESM.pdf]

Response ID	Date submitted	Date started	Date last action	The general pediatrics continuity clinic experiences for our resi	The general
3	6/16/21 13:05	6/16/21 13:00	6/16/21 13:05	located within a primary clinical site sponsored by our children's hospital or	
5	6/16/21 13:28	6/16/21 13:23	6/16/21 13:28	located within a primary clinical site sponsored by our children's hospital or	
6	6/16/21 14:14	6/16/21 14:09	6/16/21 14:14	located within a primary clinical site sponsored by our children's hospital or	
8	6/16/21 16:11	6/16/21 16:07	6/16/21 16:11	located within a primary clinical site sponsored by our children's hospital or	
9	6/17/21 9:44	6/17/21 9:32	6/17/21 9:44	A combination of the above	
10	6/17/21 21:48	6/17/21 21:45	6/17/21 21:48	located within a primary clinical site sponsored by our children's hospital or	
11	6/18/21 12:59	6/18/21 12:43	6/18/21 12:59	based in individual community preceptor practices	
13	6/28/21 15:00	6/28/21 14:56	6/28/21 15:00	based in individual community preceptor practices	
15	6/30/21 14:10	6/30/21 14:07	6/30/21 14:10	located within a primary clinical site sponsored by our children's hospital or	
18	7/14/21 18:40	7/14/21 18:37	7/14/21 18:40	located within a primary clinical site sponsored by our children's hospital or	
19	7/21/21 14:22	7/21/21 14:19	7/21/21 14:22	A combination of the above	
22	7/28/21 14:51	7/28/21 14:47	7/28/21 14:51	A combination of the above	
25	7/28/21 15:31	7/28/21 15:29	7/28/21 15:31	located within a primary clinical site sponsored by our children's hospital or	
26	7/28/21 15:54	7/28/21 15:49	7/28/21 15:54	located within a primary clinical site sponsored by our children's hospital or	
27	7/29/21 10:11	7/29/21 10:07	7/29/21 10:11	located within a primary clinical site sponsored by our children's hospital or	
30	8/18/21 15:08	8/18/21 14:42	8/18/21 15:08	A combination of the above	
31	8/25/21 11:02	8/25/21 10:58	8/25/21 11:02	located within a primary clinical site sponsored by our children's hospital or	
32	8/25/21 12:45	8/25/21 12:42	8/25/21 12:45	located within a primary clinical site sponsored by our children's hospital or	
33	8/31/21 14:06	8/31/21 13:44	8/31/21 14:06	A combination of the above	
36	3/29/22 16:05	3/29/22 16:02	3/29/22 16:05	A combination of the above	
37	3/29/22 16:08	3/29/22 16:05	3/29/22 16:08	A combination of the above	
40	3/30/22 9:37	3/30/22 9:28	3/30/22 9:37	located within a primary clinical site sponsored by our children's hospital or	
41	3/30/22 14:20	3/30/22 14:16	3/30/22 14:20	A combination of the above	
43	3/31/22 8:18	3/31/22 8:14	3/31/22 8:18	A combination of the above	
45	4/4/22 6:44	4/4/22 6:35	4/4/22 6:44	located within a primary clinical site sponsored by our children's hospital or	
46	4/4/22 9:44	4/4/22 9:37	4/4/22 9:44	A combination of the above	
49	4/6/22 12:39	4/6/22 12:36	4/6/22 12:39	other	off-site FQH
51	4/6/22 12:40	4/6/22 12:37	4/6/22 12:40	A combination of the above	
52	4/6/22 12:44	4/6/22 12:39	4/6/22 12:44	located within a primary clinical site sponsored by our children's hospital or	
53	4/6/22 12:46	4/6/22 12:43	4/6/22 12:46	located within a primary clinical site sponsored by our children's hospital or	

55	4/6/22 14:45	4/6/22 12:46	4/6/22 14:45	A combination of the above	
57	4/6/22 13:38	4/6/22 13:34	4/6/22 13:38	A combination of the above	
58	4/6/22 14:31	4/6/22 14:25	4/6/22 14:31	other	Stand alone
59	4/6/22 15:08	4/6/22 15:03	4/6/22 15:08	located within a primary clinical site sponsored by our children's hospital or	
60	4/6/22 15:34	4/6/22 15:31	4/6/22 15:34	located within a primary clinical site sponsored by our children's hospital or	
62	4/7/22 11:45	4/7/22 11:41	4/7/22 11:45	located within a primary clinical site sponsored by our children's hospital or	
63	4/8/22 11:20	4/8/22 11:15	4/8/22 11:20	A combination of the above	
4	6/16/21 13:16	6/16/21 13:11	6/16/21 13:16	located within a primary clinical site sponsored by our children's hospital or	
7	6/16/21 14:39	6/16/21 14:36	6/16/21 14:39	A combination of the above	
12	4/7/22 10:48	6/21/21 12:22	4/7/22 10:48	based in individual community preceptor practices	
14	6/29/21 7:51	6/29/21 7:45	6/29/21 7:51	located within a primary clinical site sponsored by our children's hospital or	
16	6/30/21 16:20	6/30/21 16:11	6/30/21 16:20	located within a primary clinical site sponsored by our children's hospital or	
20	7/28/21 14:41	7/28/21 14:35	7/28/21 14:41	A combination of the above	
21	7/28/21 14:47	7/28/21 14:41	7/28/21 14:47	located within a primary clinical site sponsored by our children's hospital or	
23	7/28/21 15:04	7/28/21 14:58	7/28/21 15:04	located within a primary clinical site sponsored by our children's hospital or	
28	7/29/21 22:13	7/29/21 18:12	7/29/21 22:13	other	Based in a F
29	7/30/21 7:30	7/30/21 7:26	7/30/21 7:30	located within a primary clinical site sponsored by our children's hospital or	
39	3/29/22 18:27	3/29/22 18:20	3/29/22 18:27	located within a primary clinical site sponsored by our children's hospital or	
42	3/30/22 20:42	3/30/22 20:37	3/30/22 20:42	located within a primary clinical site sponsored by our children's hospital or	
44	4/1/22 18:26	4/1/22 18:14	4/1/22 18:26	located within a primary clinical site sponsored by our children's hospital or	
47	4/4/22 16:20	4/4/22 15:05	4/4/22 16:20	A combination of the above	
50	4/6/22 13:02	4/6/22 12:37	4/6/22 13:02	A combination of the above	
54	4/6/22 12:50	4/6/22 12:45	4/6/22 12:50	A combination of the above	
56	4/6/22 13:24	4/6/22 13:18	4/6/22 13:24	A combination of the above	
61	4/6/22 19:54	4/6/22 19:18	4/6/22 19:54	located within a primary clinical site sponsored by our children's hospital or	
66	4/12/22 6:07	4/12/22 6:01	4/12/22 6:07	located within a primary clinical site sponsored by our children's hospital or	

Does your re:	At the clinic(	Do residents	At which leve	At which leve	At which leve	Does your institution have an outpat
yes	Attending ph	No	N/A	N/A	N/A	no
yes	Pediatric Res	No	N/A	N/A	N/A	yes
yes	Attending ph	No	N/A	N/A	N/A	no
yes	Pediatric Res	Yes	Yes	Yes	Yes	no
yes	Attending ph	unsure	N/A	N/A	N/A	yes
yes	Attending ph	No	N/A	N/A	N/A	yes
yes	Attending ph	No	N/A	N/A	N/A	unsure
yes	Attending ph	unsure	N/A	N/A	N/A	yes
yes	Pediatric Res	Yes	Yes	Yes	Yes	yes
yes	Attending ph	unsure	N/A	N/A	N/A	yes
yes	Pediatric Res	unsure	N/A	N/A	N/A	no
yes	Attending ph	unsure	N/A	N/A	N/A	no
yes	Attending ph	No	N/A	N/A	N/A	yes
yes	Attending ph	unsure	N/A	N/A	N/A	no
yes	Attending ph	No	N/A	N/A	N/A	yes
yes	Attending ph	Yes	Yes	Yes	Yes	no
yes	Pediatric Res	No	N/A	N/A	N/A	no
yes	Attending ph	Yes	Yes	Yes	Yes	yes
yes	Pediatric Res	unsure	N/A	N/A	N/A	yes
yes	Attending ph	No	N/A	N/A	N/A	yes
yes	Attending ph	No	N/A	N/A	N/A	yes
yes	Attending ph	unsure	N/A	N/A	N/A	no
yes	Attending ph	No	N/A	N/A	N/A	no
yes	Attending ph	No	N/A	N/A	N/A	yes
yes	Attending ph	No	N/A	N/A	N/A	no
yes	Pediatric Res	No	N/A	N/A	N/A	no
yes	Attending ph	unsure	N/A	N/A	N/A	no
yes	Advanced Pra	Yes	Yes	Yes	Yes	no
yes	Attending ph	Yes	Yes	Yes	Yes	no
yes	Pediatric Res	unsure	N/A	N/A	N/A	no

yes	Attending ph	No	N/A	N/A	N/A	no
yes	Attending ph	Yes	No	No	Yes	yes
yes	Pediatric Res	Yes	No	Yes	No	yes
yes	Attending ph	Yes	Yes	Yes	Yes	yes
yes	Pediatric Res	unsure	N/A	N/A	N/A	no
yes	Pediatric Res	Yes	Yes	Yes	Yes	no
yes	Attending ph	No	N/A	N/A	N/A	yes
yes	Pediatric Res	No	N/A	N/A	N/A	yes
yes	Pediatric Res	Yes	No	Yes	Yes	yes
yes	Attending ph	unsure	N/A	N/A	N/A	yes
yes	Pediatric Res	Yes	Yes	Yes	Yes	yes
yes	Pediatric Res	No	N/A	N/A	N/A	yes
yes	Attending ph	Yes	Yes	Yes	Yes	yes
yes	Pediatric Res	Yes	Yes	Yes	Yes	no
yes	Attending ph	No	N/A	N/A	N/A	yes
yes	Attending ph	Yes	Yes	Yes	Yes	no
yes	Attending ph	No	N/A	N/A	N/A	yes
yes	Pediatric Res	Yes	Yes	No	No	yes
yes	Attending ph	Yes	Yes	Yes	Yes	yes
yes	Attending ph	Yes	No	Yes	No	no
no		No	N/A	N/A	N/A	yes
yes	Attending ph	No	N/A	N/A	N/A	yes
yes	Attending ph	Yes	Yes	Yes	Yes	no
yes	Attending ph	No	N/A	N/A	N/A	yes
yes	Attending ph	No	N/A	N/A	N/A	no
yes	Pediatric Res	Yes	Yes	Yes	Yes	yes

Does your hospital system have a specific inpatie	Do residents	Do you provi	Aside from rotational
No		no	no
No		no	no
No		no	no
No		Yes (2-4 wee	no
No		no	no
No		no	no
No		no	no
No		Yes (2-4 wee	no
Yes	Yes, this is a	Yes (2-4 wee	no
No		Yes (2-4 wee	no
No		no	no
No		no	no
No		no	no
No		no	no
Yes	Yes, this is a	Yes (2 weeks	no
No		no	no
No		no	no
Yes	Yes, this is a	no	no
No		Yes (2-4 wee	no
No		no	no
No		Yes (2-4 wee	no
No		no	no
Yes	Yes, this is a	no	no
No		no	no
No		no	no
No		no	no
No		no	no
No		no	no
No		no	no
No		no	no

No		no	no
No		no	no
No		Yes (2-4 wee	no
No		no	no
Yes	Yes, this is a	no	no
No		no	no
No		no	no
No		Yes (2-4 wee	yes
No		no	yes
No		no	yes
Yes	No	no	yes
No		Yes (2-4 wee	yes
Yes	Yes, this is a	no	yes
No		no	yes
No		Yes (2-4 wee	yes
No		Yes (2-4 wee	yes
Yes	Yes, this is a	no	yes
No		no	yes
No		Yes (2-4 wee	yes
No		no	yes
Yes	Yes, this is a	Yes (2-4 wee	yes
No		Yes (2-4 wee	yes
No		no	yes
Yes	Yes, but offe	Yes (2-4 wee	yes
No		no	yes
Yes	Yes, this is a	Yes (2 weeks	yes

Is your curriculum: [Traditional didactics]	Is your curriculum: [Si	Is your curriculum: [Asynchronous modules/reading]
N/A	N/A	N/A
N/A	N/A	N/A
N/A	N/A	N/A
N/A	N/A	N/A
N/A	N/A	N/A
N/A	N/A	N/A
N/A	N/A	N/A
N/A	N/A	N/A
N/A	N/A	N/A
N/A	N/A	N/A
N/A	N/A	N/A
N/A	N/A	N/A
N/A	N/A	N/A
N/A	N/A	N/A
N/A	N/A	N/A
N/A	N/A	N/A
N/A	N/A	N/A
N/A	N/A	N/A
N/A	N/A	N/A
N/A	N/A	N/A
N/A	N/A	N/A
N/A	N/A	N/A
N/A	N/A	N/A
N/A	N/A	N/A
N/A	N/A	N/A
N/A	N/A	N/A
N/A	N/A	N/A
N/A	N/A	N/A
N/A	N/A	N/A
N/A	N/A	N/A

N/A	N/A	N/A
N/A	N/A	N/A
N/A	N/A	N/A
N/A	N/A	N/A
N/A	N/A	N/A
N/A	N/A	N/A
N/A	N/A	N/A
yes	No	No
yes	No	No
yes	No	No
yes	yes	yes
No	No	yes
yes	yes	yes
yes	No	yes
No	No	yes
No	No	yes
yes	No	No
yes	No	yes
yes	yes	yes
yes	No	yes
yes	yes	No
yes	No	yes
yes	No	No
yes	yes	yes
yes	No	No
yes	No	yes

Is your curriculum: [Based on experiential	Is your curric	Please descri	When residents gradua	Aside from time, what are fa
N/A			very prepared	No
N/A			very prepared	No
N/A			very prepared	yes
N/A			very prepared	No
N/A			extremely prepared	No
N/A			somewhat prepared	yes
N/A			somewhat prepared	No
N/A			somewhat prepared	yes
N/A			very prepared	No
N/A			somewhat prepared	No
N/A			very prepared	No
N/A			somewhat prepared	yes
N/A			very prepared	No
N/A			somewhat prepared	yes
N/A			somewhat prepared	yes
N/A			somewhat prepared	yes
N/A			extremely prepared	No
N/A			somewhat prepared	yes
N/A			somewhat prepared	yes
N/A			somewhat prepared	No
N/A			somewhat prepared	yes
N/A			very prepared	yes
N/A			very prepared	yes
N/A			somewhat prepared	No
N/A			slightly prepared	yes
N/A			somewhat prepared	yes
N/A			somewhat prepared	No
N/A			somewhat prepared	No
N/A			somewhat prepared	No
N/A			somewhat prepared	No

N/A			not at all prepared	yes
N/A			extremely prepared	yes
N/A			very prepared	yes
N/A			somewhat prepared	No
N/A			very prepared	yes
N/A			very prepared	No
N/A			somewhat prepared	No
No		We are still	very prepared	No
No		It is brief. W	very prepared	No
No		The resident	somewhat prepared	yes
No		Every month	very prepared	yes
yes	Flipped class	We offer an	very prepared	No
No		We have noc	somewhat prepared	yes
yes		As part of ou	very prepared	No
yes		This is a 4 w	extremely prepared	No
No	Continuity ta	We go over	somewhat prepared	No
yes		When on cor	extremely prepared	No
yes		Xxx	somewhat prepared	No
yes		The curriculu	extremely prepared	yes
yes		Our resident	very prepared	yes
yes		Simulation:	very prepared	No
yes		We have a pediatric	somewhat prepared	yes
No		Integrated in	very prepared	No
yes		For those res	very prepared	No
yes		1 dedicated l	somewhat prepared	No
No		there is no s	somewhat prepared	yes

Aside from time, what are	Aside from time, what are	Aside from time, what ar	Aside from time, what a
yes	No	No	No
yes	No	yes	No
No	No	yes	No
yes	yes	No	No
No	No	No	No
No	yes	yes	No
No	No	No	No
No	yes	No	No
No	No	No	No
No	No	yes	No
No	No	No	No
yes	yes	yes	No
No	yes	yes	No
No	No	No	yes
yes	No	yes	No
yes	yes	No	No
yes	No	No	No
No	No	yes	No
No	yes	No	yes
yes	yes	yes	No
No	No	No	No
No	yes	No	No
No	yes	No	No
No	No	No	yes
No	No	yes	No
No	yes	No	No
No	yes	yes	No
No	No	No	No
No	No	No	No
No	No	No	yes

yes	No	yes	yes
yes	No	No	No
No	yes	No	No
yes	yes	yes	No
yes	yes	No	No
yes	yes	No	No
No	No	No	No
yes	yes	No	No
No	No	No	No
No	No	No	yes
No	yes	No	No
yes	yes	No	No
No	yes	No	No
No	yes	No	No
No	No	No	No
No	No	yes	No
No	yes	No	No
No	No	No	No
yes	yes	No	No
No	yes	yes	No
yes	yes	yes	No
No	No	No	No
yes	yes	No	No
yes	No	yes	No
No	yes	yes	No
No	No	No	yes

Aside from time, what are factors	Aside from time, what are fac	Aside from time, what are factors that limit resident pr	In a recent De
No	No		3
No	No		3
yes	No		2
No	No		4
yes	No		4
No	No		4
No	No	I am not sure I can say how prepared they feel or the fa	3
No	No	lack of time in program carved out to this area	3
yes	No		4
yes	yes		3
No	No	unknown	4
No	No		3
No	No		4
yes	No		3
No	yes		3
No	No	varying experiences in community vs academic based co	3
yes	No		4
No	yes		4
No	No		3
yes	yes		3
No	No		3
yes	No		3
No	No		4
No	yes	I think low volume in that the kids with CMC need things	4
yes	No		1
No	No		3
No	yes		3
No	yes		4
No	No	Residents don't uniformly take ownership of medically c	4
yes	No		4

yes	yes		3
yes	No		5
No	yes		4
No	No		3
No	No		4
yes	No		4
yes	No		3
No	yes		3
No	No		4
yes	No		4
yes	No		4
No	No	Residents are not as involved with the non clinical aspe	4
yes	No		5
No	No		4
No	No	None of these are an issue	5
No	yes	patient no show rates , central scheduling inconsistent	
No	No		5
No	No	They are at least as prepared for cmc as any other child	5
No	No		5
No	No		3
No	No		4
No	No		3
No	No		5
yes	No		4
No	No		3
yes	No		3

No curriculum	3.41
Curriculum	4.06

0.72493139
0.80236578

In a recent De	In a recent De	In a recent De	In a recent De	In a recent De	In a recent De	In a recent De	In a recent De	In a recent De
3	3	3	3	3	4	3	3	4
3	4	3	3	4	4	3	3	2
3	2	3	3	4	4	4	4	3
3	3	3	3	4	4	4	3	3
4	3	4	4	4	4	4	3	4
4	2	1	3	3	3	3	4	3
Unsure	1	2	3	4	2	3	2	Unsure
3	4	3	4	3	4	4	3	4
4	4	4	3	4	4	4	4	4
2	2	2	2	2	3	2	2	Unsure
4	3	4	4	5	4	4	3	3
3	3	3	Unsure	2	2	3	Unsure	Unsure
3	3	3	4	4	4	3	1	2
2	3	3	3	4	3	2	1	2
3	1	3	3	3	3	4	3	3
3	3	4	4	3	4	3	3	3
4	2	4	4	5	5	5	4	3
3	4	3	2	3	3	4	3	3
3	2	2	3	3	3	3	2	3
4	2	2	3	4	2	4	3	2
3	1	2	1	2	2	2	1	1
3	1	4	3	4	3	4	1	2
3	2	3	3	5	4	4	3	3
3	3	3	3	4	4	3	3	3
1	1	1	1	1	1	1	1	1
3	3	3	2	4	4	4	3	3
3	3	3	3	3	3	3	3	3
4	3	3	3	4	5	4	4	5
3	3	4	3		4	Unsure	Unsure	Unsure
4	3	4	4	5	3	4	3	4

4	2	1	1	3	4	2	2	2
3	3	5	5	5	4	5	4	4
3	2	3	3	4	3	4	2	3
4	3	3	3	3	3	3	3	3
3	3	3	4	4	3	3	3	3
3	3	4	3	4	4	4	3	3
4	3	4	3	3	4	4	Unsure	3
3	4	4	3	5	4	4	3	3
3	4	4	3	4	3	3	3	3
3	2	3	3	3	3	3	4	3
3	4	4	3	4	4	3	3	3
3	3	4	3	2	4	3	3	4
5	2	3	4	5	5	4	3	2
3	4	5	4	4	5	4	3	4
2	5	5	5	5	4	5	2	3
4	5	5	4	5	5	5	5	4
3	3	4	4	4	3	4	3	3
5	4	5	5	5	5	5	5	4
3	3	4	3	4	3	3	3	3
4	3	4	3	5	4	5	3	3
3	2	3	3	4	3	3	3	3
3	3	3	3	5	4	4	3	2
3	4	5	4	4	3	3	2	3
2	3	2	2	3	2	2	3	2
3	3	3	3	3	4	4	3	3

3.19	2.59	3.03	3.03	3.58	3.43	3.42	2.74	2.94
3.22	3.39	3.89	3.44	4.11	3.78	3.72	3.17	3.06

0.66845	0.86472409	0.92755612	0.87785814	0.93732141	0.86732477	0.87423436	0.9312365	0.8638357
0.80845208	0.91644382	0.90025414	0.78382338	0.90025414	0.87820375	0.89479249	0.78590525	0.63913749

In a recent De	Please indica	If not mandated by the ACGME, how likely are yo	Why are you slightly likely or not at all	Should childr
3	3	not at all likely	Why this man to say for curriculum ch	no
4	4	slightly likely	Clinical and lecture time constraints, t	Yes
3	5	slightly likely	Competing educational interests acros	Yes
4	5	very likely		Yes
3	5	very likely		Yes
3	5	somewhat likely		Yes
2	4	somewhat likely		Yes
4	5	extremely Likely		Yes
4	5	somewhat likely		Yes
3	5	very likely		Yes
4	5	very likely		Yes
2	5	extremely Likely		Yes
3	4	very likely		unsure
2	5	somewhat likely		Yes
4	1	very likely		Yes
3	5	very likely		Yes
2	5	somewhat likely		Yes
5	4	very likely		Yes
3	3	not at all likely	We already tried this years ago with [I	Yes
3	4	slightly likely	time and implementation barriers (res	unsure
2	5	very likely		Yes
4	5	somewhat likely		Yes
4	5	somewhat likely		Yes
4	5	somewhat likely		Yes
1	5	slightly likely	Need to find faculty who can teach it	no
3	5	somewhat likely		Yes
3	5	very likely		Yes
4	4	somewhat likely		Yes
Unsure	4	very likely		unsure
5	5	slightly likely	Time and resource availability	Yes

2	5	very likely		no
5	5	extremely Likely		Yes
2	4	very likely		Yes
3	5	very likely		Yes
3	5	n/a: Our program already has a formal curriculum		Yes
3	4	somewhat likely		Yes
3	4	slightly likely	There are so many requirements and r	Yes
4	5	n/a: Our program already has a formal curriculum		Yes
4	5	somewhat likely		Yes
2	4	somewhat likely		Yes
4	4	very likely		Yes
3	5	n/a: Our program already has a formal curriculum		Yes
4	3	slightly likely	Competing educational demands that	Yes
5	5	somewhat likely		Yes
5	5	n/a: Our program already has a formal curriculum		Yes
	4	somewhat likely		Yes
5	5	n/a: Our program already has a formal curriculum		Yes
4	5	n/a: Our program already has a formal curriculum		Yes
5	5	very likely		Yes
3	5	very likely		unsure
5	5	extremely Likely		Yes
3	5	n/a: Our program already has a formal curriculum		unsure
3	5	somewhat likely		Yes
4	5	very likely		Yes
3	5	somewhat likely		Yes
3	5	very likely		unsure

3.19
3.83

0.95077245
0.92354815

Please place	Region	Program Setting	Total Reside	Categorical Residents
WE must tea	Southeast	Community-based, university-affiliated	68	65
n/a	Mid-America	University-based	27	27
	Southwest	Military	40	40
	Western	University-based	40	40
	Southeast	Community-Based,Community-based, university-affiliated	84	84
	New York	Community-Based,Community-based, university-affiliated	57	53
This is a toug	Mid-America	Community-Based	62	62
	Southeast	Community-based, university-affiliated	41	37
	Southeast	Community-Based,Community-based, university-affiliated	31	31
	Mid-America	Community-Based,Community-based, university-affiliated	12	12
N/A	Western	University-based	48	48
	New York	Community-Based,Community-based, university-affiliated	47	33
	Mid-America	University-based	79	75
n/A	Mid-America	Community-Based,Community-based, university-affiliated	60	60
N/A	Western	University-based	84	84
we have a bl	Midwest	Community-Based,Community-based, university-affiliated	43	43
	Southeast	University-based	90	71
	New York	University-based	46	46
Some of the	Western	University-based	101	71
It takes WAY	Northeast	Community-Based,Community-based, university-affiliated	40	34
	Southeast	Community-Based,Community-based, university-affiliated	18	18
In many sma	New York	Community-Based	54	54
	Western	University-based	128	121
	Northeast	Community-based, university-affiliated	64	42
For the child	New York	University-based	44	44
Super import	Southeast	Community-Based	35	35
	Mid-Atlantic	Community-Based,Community-based, university-affiliated	16	16
	New York	Community-Based,Community-based, university-affiliated	18	18
	Mid-Atlantic	Community-Based,Community-based, university-affiliated	24	24
	Western	Community-Based,Community-based, university-affiliated	23	23

Thank you fo	New York	Community-Based,Community-based, university-affiliated	43	43
In addition tc	Southeast	Community-Based,Community-based, university-affiliated	23	23
	Southwest	University-based	24	24
	Western	Community-Based,Community-based, university-affiliated	82	80
	Southeast	University-based	52	52
	Western	University-based	83	79
	Western	University-based	56	52
	Southwest	University-based	38	38
	New York	Community-Based,Community-based, university-affiliated	60	60
	Midwest	University-based	101	87
	Southeast	University-based	35	33
It is importa	Southwest	University-based	40	40
	Northeast	University-based	165	124
	Northeast	Community-Based,Community-based, university-affiliated	18	18
	Southwest	Community-Based,Community-based, university-affiliated	39	39
I feel strongl	Western	Community-Based	8	8
We also have	Mid-Atlantic	Community-Based,Community-based, university-affiliated	159	126
It is rare for	Western	Community-Based,Community-based, university-affiliated	24	24
	Midwest	University-based	52	48
I think reside	New York	Community-based, university-affiliated	79	77
	Southwest	University-based	65	63
	Midwest	University-based	46	46
	Mid-Atlantic	Community-based, university-affiliated	39	42
	Western	Community-based, university-affiliated	38	38
	Southwest	Community-Based,Community-based, university-affiliated	21	21
	Southeast	University-based	46	46

Program Size
M
S
M
M
L
M
M
M
M
S
M
M
L
M
L
M
L
M
L
M
S
M
L
M
M
M
S
S
S
S

M
S
S
L
M
L
M
M
M
L
M
M
L
S
M
S
L
S
M
L
M
M
M
M
S
M
